# Contrast‐Enhanced CT Finding of Hot Spot Sign Secondary to Supra Hilar Mass Causing Superior Vena Cava Syndrome: A Case Report

**DOI:** 10.1002/ccr3.73558

**Published:** 2026-09-28

**Authors:** Ghazala Shahzad, Rabail Memon, Syed Muhammad Salman Hassan, Fatima Khan, Remsha Hammeed Khan, Muddassir Khalid, Fred Segawa

**Affiliations:** ^1^ Liaquat University of Medical and Health Sciences Jamshoro Pakistan; ^2^ Nishtar Medical University Multan Pakistan; ^3^ Islamabad Medical and Dental College Islamabad Pakistan; ^4^ Makerere University College of Health Sciences Kampala Uganda

**Keywords:** contrast‐enhanced CT scan, hot spot sign, supra‐hilar mass, SVC syndrome, vascular liver diseases

## Abstract

Superior vena cava syndrome is caused by obstruction of the superior vena cava leading to impaired drainage of the head, neck, and upper chest. A hot spot sign is a rare but sensitive radiological finding and is described as a focal area of hyper‐enhancement in Segment IVa (quadrate lobe) of the liver. It demonstrates an area of increased blood flow due to a collateral venous pathway in the setting of superior vena cava obstruction. We report a 60‐year‐old male presenting with facial and upper limb swelling, dyspnea, cough, weight loss, and prominent anterolateral chest wall veins. Laboratory tests were unremarkable. Contrast‐enhanced computed tomography (CT) revealed a right suprahilar mass infiltrating the SVC and azygos vein, with dense opacification of collateral pathways and a hyper‐enhancing wedge‐shaped area in Segment IVa of the liver, consistent with the “hot spot sign.” Bronchoscopy and biopsy confirmed lung adenocarcinoma, positive for TTF‐1 and negative for P40. The hepatic hot spot sign arises from portosystemic shunting via the superior vein of Sappey, which connects systemic veins—including phrenic, internal thoracic, and superior epigastric veins—to the left portal vein. Recognition of this pseudo‐lesion is critical, as it may mimic metastatic liver lesions and prevent unnecessary invasive procedures such as liver biopsy. Early identification of SVC obstruction facilitates prompt management and prevents complications. Contrast‐enhanced CT plays a pivotal role in detecting vascular liver changes secondary to SVC obstruction. Awareness of the hot quadrate sign is essential for early diagnosis and appropriate management of underlying malignancy.

## Key Clinical Message

The hepatic hot quadrate sign on contrast‐enhanced CT indicates collateral venous flow secondary to superior vena cava obstruction. Early recognition prevents misdiagnosis as liver metastasis, guides timely investigation of the underlying cause—often malignancy—and avoids unnecessary invasive procedures like liver biopsy.

## 1 Introduction

Superior vena cava (SVC) syndrome most commonly arises from extrinsic compression of the vessel by malignant processes, including bronchogenic carcinoma, lymphoma, and metastatic mediastinal lymphadenopathy [1]. Benign etiologies include fibrosing mediastinitis, granulomatous mediastinal disease, aortic aneurysms, retrosternal goiter, intrinsic thrombosis related to prothrombotic states, and iatrogenic factors such as central venous catheterization or transvenous pacemaker placement [1].

Clinically, SVC syndrome is characterized by venous congestion of the upper body, resulting in facial and upper limb edema, dilated superficial veins, cyanosis, and respiratory compromise due to edema of airway structures [1]. When venous obstruction develops gradually or remains partial, extensive collateral circulation may form, rendering symptoms minimal or clinically unapparent [1]. In such cases, the development of collateral venous pathways may precede clinical manifestations [1]. These collateral channels play a critical role in SVC syndrome diagnosis, as they give rise to characteristic imaging findings that provide clues to underlying SVC obstruction [1].

A rare but highly instructive manifestation of collateralization is “hot spot” sign, first described in 1983 by Ishikawa [2]. The sign can be observed on technetium‐99 m (^99m^T_C_) Sulfur colloid scan or on contrast‐enhanced computed tomography (CT) scans [2, 3]. On contrast enhanced CT the hot spot sign appears as a localized wedge‐shaped hyperdense area in Segment IVa of the liver that results from portosystemic venous shunting between the superior vena cava and the portal venous system [4]. The enhancement occurs due to rerouted venous flow through collateral pathways; elevated systemic venous pressure forces blood backward through the internal thoracic and phrenic veins into the superior vein of Sappey, which directly drains into the left portal vein branch supplying Segment IVa. This localized rush of contrast‐rich systemic blood during early imaging phases creates a hypervascular pseudolesion that may mimic a focal hepatic tumor or metastasis [3]. Despite its value in identifying Superior vena cava obstruction, this sign remains underreported in the clinical literature [3]. We present a case of the CT hot spot sign, caused by SVC obstruction duce to infiltrating adenocarcinoma of lung and discuss its physiological basis and importance. Recognizing it early is important to prevent further invasive procedures such as liver biopsy. This case report has been reported in line with CARE checklist.

## 2 Case Presentation

A 60 years old male, smoker with no significant past medical history presented to pulmonary OPD in Hyderabad with the history of chest pain, productive cough with few episodes of hemoptysis for 2 months, shortness of breath, dizziness and significant unexplained weight loss. Clinical examinations show facial and arm swelling, facial redness and prominent, dilated veins in chest and upper abdomen along with raised non‐pulsatile jugular venous pressure. Chest auscultation revealed reduced entry of air in the right upper zone. Baseline laboratory investigations were within the normal limits.

A contrast enhanced CT scan was performed for suspected superior vena cava obstruction; a 64‐slice MDCT scanner was utilized. Contrast‐enhanced CT was performed following the intravenous administration of 100–150 mL of non‐ionic iodinated contrast agent via a right antecubital vein at an injection rate of 4.0 mL/s. Imaging acquisition of the thorax and upper abdomen was captured in the early arterial phase, approximately 15–25 s post‐injection. An irregular supra‐hilar region mass was identified infiltrating and obstructing the superior vena cava and azygous vein causing its luminal narrowing and occlusion, manifesting as extensive collaterization. Notably, superficial and deep collaterals were observed including internal thoracic and phrenic vein.

The obstruction causes increased systemic venous pressure. Vein of sappey flows within the upper falciform ligament and constitutes a shunt between the systemic venous system including phrenic, internal thoracic as well as the superior epigastric veins with portal venous subdivisions of left hepatic lobe mainly Segment IVa. In the presence of elevated systemic venous pressure, this vein may function as a collateral channel for shunting of systemic venous blood into the portal circulation. This focal alternation resulted in a definite hepatic hot spot sign, visible as an intense localized area of focal hyperenhancement in Segment IVa of liver.

To establish a definitive pathological diagnosis, the patient underwent flexible bronchoscopy, which revealed an exophytic endobronchial mass obstructing the apical and anterior segments of the right upper lobe. Endobronchial biopsy specimens demonstrated sheets and nests of atypical epithelial cells with hyperchromatic, enlarged nuclei and prominent nucleoli. On immunohistochemical staining, the tumor cells demonstrated strong diffuse positivity for thyroid transcription factor‐1 (TTF‐1) and were completely negative for p40. These histopathological features confirmed a primary lung adenocarcinoma.

### 2.1 Differential Diagnosis of Segment IVa Lesions

In order to differentiate this benign vascular perfusion variant from actual space‐occupying focal hepatic lesions, it is important to recognize the hepatic hot spot sign. It can readily resemble hypervascular diseases on typical CT profiles (Table 1).

**TABLE 1:** Differential diagnoses of Segment IVa lesions.

Pathology	Location and morphology	Enhancement pattern	Distinguishing features
**Hepatic Hot Spot Sign**	Strictly Segment IVa; characteristically **wedge‐shaped** in quadrate without mass effect	Intense, homogeneous hyperenhancement during early phases; becomes **isodense** on delayed phases	Absence of structural distortion; directly associated with thoracic SVC obstruction and thoracoabdominal collaterals
**Hypervascular Metastasis** (e.g., neuroendocrine, renal cell carcinoma)	Variable, multiple, random distribution throughout the liver; distinct **rounded mass**	Early peripheral rim enhancement with rapid centripetal progression or washout	Exhibits mass effect, distorts adjacent vessels, and often demonstrates an regular contour or necrotic center
**Hepatic Hemangioma**	Variable distribution; sharply demarcated, rounded or lobulated	Discontinuous, peripheral **nodular** enhancement with gradual centripetal fill‐in on delayed phases	Persistent enhancement on delayed imaging without wash‐out; independent of central venous pressures
**Focal Nodular Hyperplasia (FNH)**	Variable; well‐circumscribed solitary mass	Homogeneous intense arterial enhancement, often sparing a **central scar**	The central scar remains hypoattenuating in early phases and fills in on delayed images; no associated systemic collateral tracks
**Pyogenic Hepatic Abscess**	Variable; round, thick‐walled fluid collection	“Double‐target” sign; rim‐enhancement surrounding a central non‐enhancing fluid cavity	Accompanied by peripheral edema, clinical sepsis, and leukocytosis rather than signs of SVC syndrome

### 2.2 Investigations

A contrast enhanced CT scan was performed; a supra‐hilar region mass was identified. It was seen infiltrating the superior vena cava and azygous vein, resulting in increased systemic venous pressure. Vein of sappey flows within the upper falciform ligament and constitutes a shunt between the systemic venous system including phrenic, internal thoracic as well as the superior epigastric veins with portal venous subdivisions of left hepatic lobe mainly Segment IVa. In the presence of elevated systemic venous pressure, this vein may function as a collateral channel for shunting of systemic venous blood into the portal circulation. A hepatic pseudo‐lesion or “hot spot,” focal hyper‐enhancement of Segment IVa on contrast‐enhanced images may be observed due to this interplay of hemodynamics (Figures 1 and 2).

**FIGURE 1:**
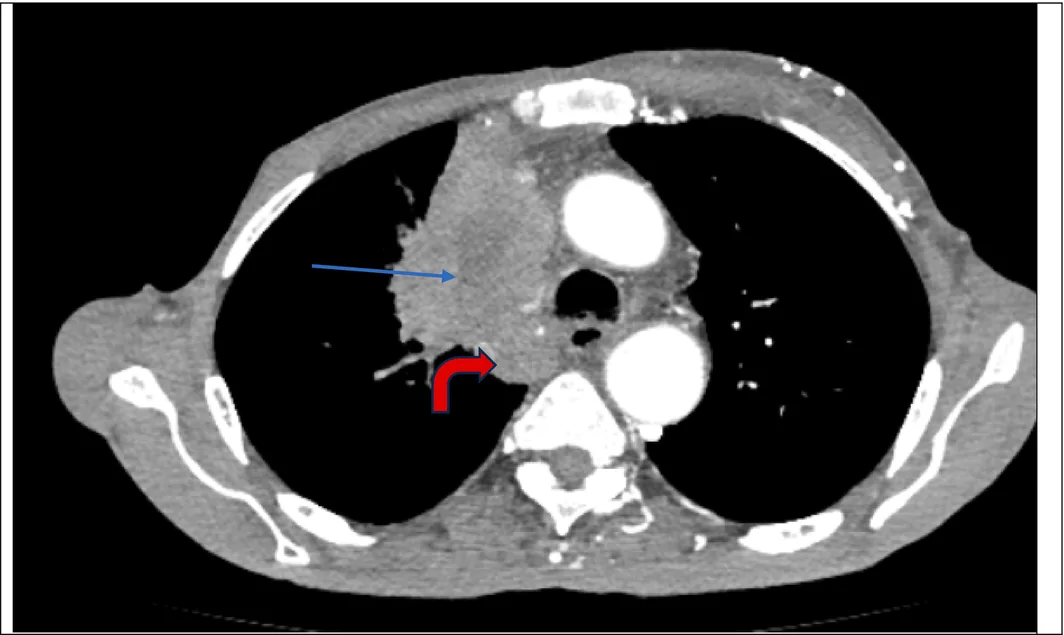
Axial contrast‐enhanced CT image of the chest. The long blue arrow demonstrates the primary right suprahilar mass. The red arrow highlights the direct tumor invasion and occlusion of the lumen of the superior vena cava.

**FIGURE 2:**
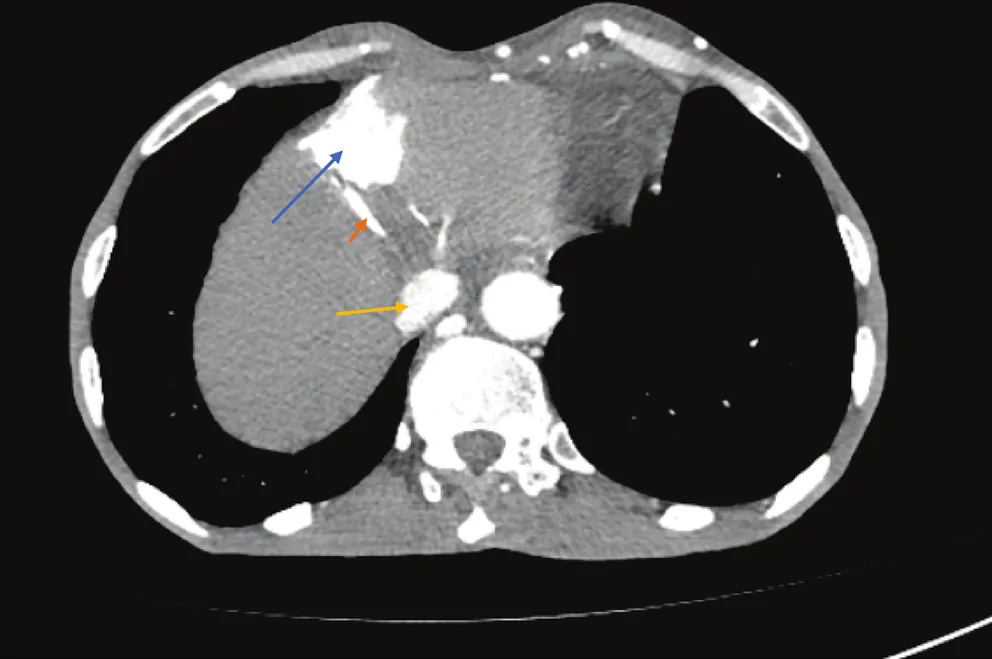
Axial contrast‐enhanced CT image of the upper abdomen. The long blue arrow identifies the classic hepatic hot spot sign, seen as localized hyper‐enhancement within Segment IVa (quadrate lobe) of the liver. The short orange arrow demonstrates the middle hepatic vein, and the yellow arrow points to the dense opacification of the inferior vena cava resulting from collateral systemic reflux.

Further, Bronchoscopy was performed and biopsy was done, it revealed endobronchial mass obstructing the apical and anterior segment of right upper lobe, the lining cells are hyperchromatic and enlarged and are positive for TTf 1 and negative for P40. Features are in favor of adenocarcinoma of lung origin (Figures 3 and 4).

**FIGURE 3:**
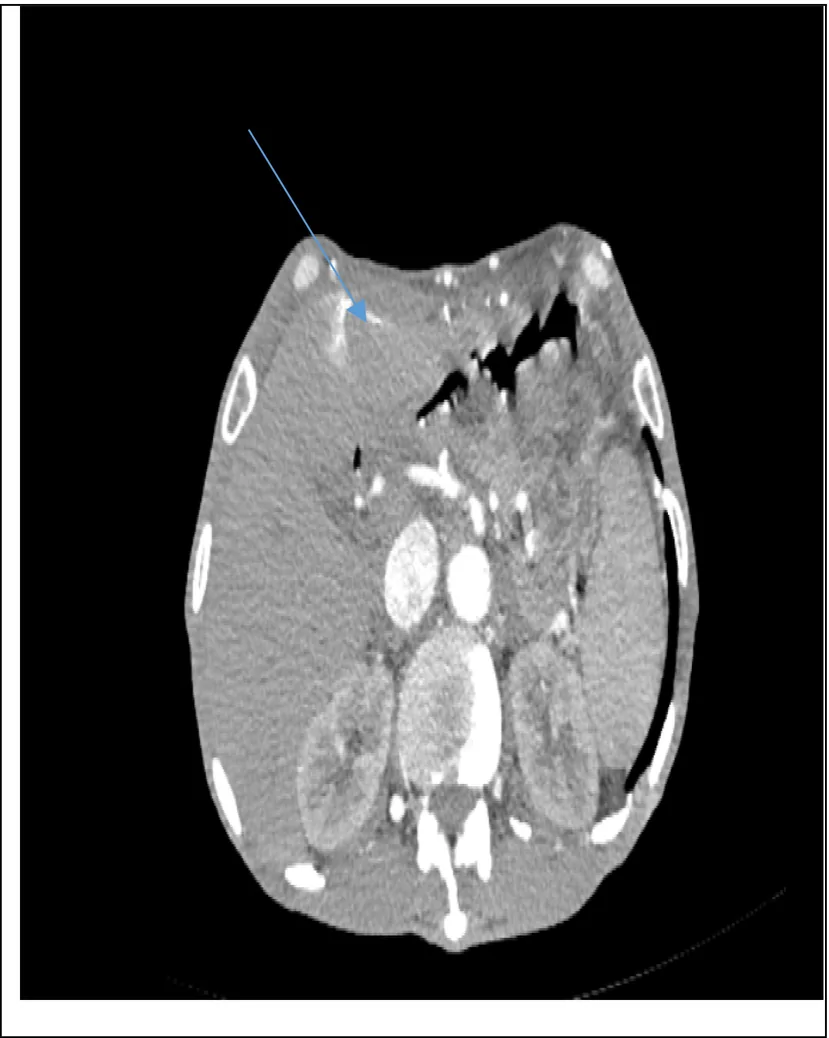
Axial section of contrast CT: long arrow points vein of sapey connecting the internal thoracic, pherenic to portal vein (not seen in this figure).

**FIGURE 4:**
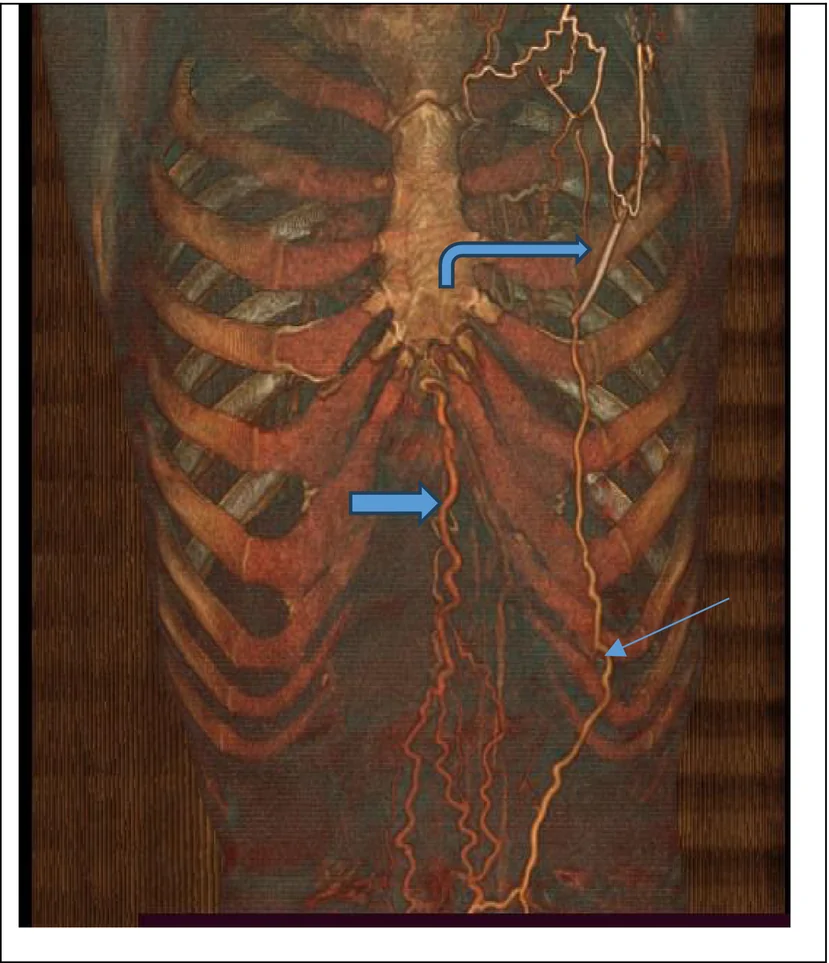
Superficial tributaries of internal mammary vein—arrow bent superficial tributaries of paraumbilical vein—long arrow. Superficial epigastric vein—block arrow.

In our case there is adenocarcinoma of lung infiltrating the superior vena cava; hot spot sign in liver is seen with dense opacification of inferior vena cava and azygous vein (Figures 1 and 2).

### 2.3 Management

The patient was evaluated by a multidisciplinary team comprising pulmonology, medical oncology, radiation oncology, and interventional radiology. Initial management of Superior vena cava syndrome consisted of head elevation, supplemental oxygen as needed, and close monitoring for airway compromise and hemodynamic instability.

Definitive management focused on the underlying malignancy:

- Tissue diagnosis: bronchoscopy‐guided biopsy confirmed right upper lobe adenocarcinoma.
- Oncologic treatment: the patient was referred to medical oncology for systemic chemotherapy and consideration for radiotherapy targeting the suprahilar mass to relieve SVC obstruction.
- Vascular support: endovascular stenting of the SVC was considered to alleviate venous congestion if symptomatic deterioration occurred and therefore was not performed during the index hospitalization.

## 3 Discussion

Superior vena cava (SVC) syndrome is a medical emergency caused by the obstruction of blood flow through the SVC, which can be fatal if left untreated [5]. Approximately 80% of cases have malignant origins—specifically bronchogenic carcinomas, sarcomas, and lymphomas. The remaining 20% stem from benign causes, including fibrosing mediastinitis, infections (such as tuberculosis and histoplasmosis), aneurysms, vasculitis, arteriovenous fistulas, mass effects from benign mediastinal tumors, and thrombosis associated with central venous catheter use [6].

The hepatic hot spot sign is a critical radiological phenomenon that acts as a beacon for central thoracic venous obstruction. Understanding the specific venous macro‐anatomy of the “third liver inflow system” is necessary to interpret this sign correctly. While the liver relies primarily on the portal vein (80%) and hepatic artery (20%) for its blood supply, accessory venous pathways—such as the parabiliary system, the cholecystic veins, and the epigastric‐paraumbilical system—can directly enter the hepatic parenchyma [7]. Third inflow of liver denotes accessory vein draining into the liver by bypassing the portal vein. The paraumbilical vein is divided into the vein of Burow, superior vein of Sappey, and inferior vein of Sappey [7]. The superior vein of Sappey traverses through the falciform ligament and naturally bridges the superior phrenic and internal thoracic (mammary) veins with the peripheral branches of the left portal vein that supply Segment IVa. When there is superior vena cava obstruction, there is increased systemic venous pressure; this acts as an alternate venous pathway towards the liver, causing hyperenhancement in Segment IVa in liver [8].

Under normal physiological conditions, flow through these tiny accessory veins is negligible. However, when a primary malignancy (such as the right suprahilar lung adenocarcinoma presented here) causes high‐grade mechanical obstruction or thrombosis of the SVC, pressure in the brachiocephalic and internal thoracic venous systems increases dramatically. This pressure gradient forces systemic venous blood to flow retrogradely down the internal thoracic and phrenic veins, through the vein of Sappey, and straight into the left portal venous system. Because this blood contains a highly concentrated bolus of contrast medium newly injected into the upper extremity, Segment IVa enhances intensely and prematurely relative to the rest of the liver parenchyma, which awaits delayed contrast arrival via the normal portal circuit.

## 4 Conclusion

Pseudo‐lesion mimic liver metastasis so in order to avoid misdiagnosis it is important to recognize hepatic hot spot sign and to early reach its primary cause secondary to superior vena cava obstruction. CECT is significant in detecting vascular liver changes secondary to SVC obstruction. Awareness of the hot quadrate sign is essential for early diagnosis and appropriate management of underlying malignancy.

## Author Contributions

**Ghazala Shahzad:** conceptualization, methodology, writing – original draft. **Rabail Memon:** writing – original draft, investigation. **Syed Muhammad Salman Hassan:** validation, visualization. **Fatima Khan:** software, writing – original draft. **Remsha Hammeed Khan:** writing – original draft. **Muddassir Khalid:** writing – review and editing, project administration, supervision. **Fred Segawa:** resources.

## Funding

The authors have nothing to report.

## Ethics Statement

Ethical approval is not required for case reports at our institution.

## Consent

Written informed consent was obtained from the patient for publication of this case report and accompanying images. A copy of the written consent is available for review by the Editor‐in‐Chief of this journal on request.

## Conflicts of Interest

The authors declare no conflicts of interest.

## Data Availability

Data available on request from the authors.
